# Development of Nanomaterial-Modified Impedimetric Aptasensor—A Single-Step Strategy for 3,4-Methylenedioxymethylamphetamine Detection

**DOI:** 10.3390/bios12070538

**Published:** 2022-07-20

**Authors:** Shringika Soni, Utkarsh Jain, Donald H. Burke, Nidhi Chauhan

**Affiliations:** 1 Amity Institute of Nanotechnology (AINT), Amity University Uttar Pradesh (AUUP), Sector-125, Noida 201313, Uttar Pradesh, India; shringika.soni@s.amity.edu (S.S.); ujain@amity.edu (U.J.); 2 Department of Molecular Microbiology and Immunology, Department of Biochemistry, and Bond Life Sciences Center, University of Missouri, Columbia, MO 65211, USA; burkedh@missouri.edu

**Keywords:** 3,4-methylenedioxymethylamphetamine, electrochemical sensor, aptamer, tin nanoparticles, recreational drugs, forensic

## Abstract

Developing rapid, sensitive detection methods for 3,4-Methylenedioxymethylamphetamine (MDMA) is crucial to reduce its current misuse in the world population. With that aim, we developed an aptamer-modified tin nanoparticle (SnNP)-based nanoarchitecture as an electrochemical sensor in this study. This platform exhibited a high electron transfer rate with enhanced conductivity arising from its large surface area in comparison to the bare electrode. This observation was explained by the 40-fold higher electroactive surface area of SnNPs@Au, which provided a large space for 1.0 μM *Apt_MDMA_* (0.68 ± 0.36 × 10^12^ molecule/cm^2^) immobilization and yielded a significant electrochemical response in the presence of MDMA. Furthermore, the *Apt_MDMA_*-modified SnNPs@Au sensing platform proved to be a simple yet ultrasensitive analytical device for MDMA detection in spiked biological and water samples. This novel electrochemical aptasensor showed good linearity in the range of 0.01–1.0 nM for MDMA (R^2^ = 0.97) with a limit of detection of 0.33 nM and a sensitivity of 0.54 ohm/nM. In addition, the device showed high accuracy and stability along with signal recoveries in the range of 92–96.7% (Relative Standard Deviation, RSD, 1.1–2.18%). In conclusion, the proposed aptasensor developed here is the first to combine SnNPs and aptamers for illicit compound detection, and it offers a reliable platform for recreational drug detection.

## 1. Introduction

The compound 3,4-Methylenedioxymethylamphetamine (MDMA) is a drug that falls under the category of amphetamine-type stimulants (ATS). It was first synthesized in 1912 by Merck as an appetite suppressant [1]. To date, there are no recognized medical applications of MDMA; however, due to its entactogenic action, MDMA is being investigated for potential use in psychiatry in the treatment of social anxiety symptoms, such as lack of communication and low empathy [2,3,4]. It is structurally similar to mescaline and amphetamine, which promote hallucinogenic and stimulant effects, respectively [5,6] and, ultimately increase drug misuse. Additionally, MDMA is reported as a major recreational drug with a high risk of depression, insomnia, impulsive behavior, irritability, and impaired cognition which may lead to fatal arrhythmia and ultimately cause death.

MDMA quantification for monitoring abuse primarily relies upon whole blood and urine specimens, which are analyzed under optimized laboratory conditions mainly via Gas Chromatography (GC) and High-Pressure Liquid Chromatography (HPLC) [7,8]. Classical techniques, such as suspended droplet-based liquid–liquid extraction and supported liquid extraction, require incubation, washing, and separation during sample processing. These techniques to functionally quantify analytes, however, are expensive and time-consuming, primarily because of the complexity of preliminary sample preparation techniques [9,10]. To broadly enable the analysis, the developed sensor for drug testing should be simple, rapid, and cost-effective.

Recently, aptamers (*Apt*) have emerged as an area of interest and excellent alternatives to chromatography and electrophoresis for active identification [11,12]. They are oligonucleotide-based affinity probes that exhibit advantages of higher selectivity, stability, and sensitivity over antibodies [13,14]. Additional superior attributes of *Apt* include the relative simplicity through which nucleic acids can be engineered in vitro to integrate affinity and signal-transducing properties into a single moiety. These properties of *Apt* make them attractive in analytical chemistry, where they identify the target via hydrogen bonding, van der Waals, or electrostatic interactions. They provide an excellent platform for sensor development (such sensors are termed ‘aptasensors’) due to their easy fabrication, customizable modification, and ultraselective detection properties. However, a single family of *Apt* can exhibit affinity for chemically related targets, such as structural or chemical analogs of the original selection target, and this cross-recognition can be exploited in the development of sensors. Additionally, the introduction of hydrophobic moieties into aptamers also expands the diversity of interactions between aptamers and targets [15].

Electrochemical sensors work on the principle of electron transfer between modified sensing surfaces and electrolytes, in which redox reactions are evaluated to confirm device development [16,17]. This simple measurement procedure improves the quantitative detection of the target analyte [18]. Once an aptamer-modified surface is fabricated, a decrease in the current is observed due to the non-conducting nature of the biological entity [19,20]. Further conformational changes in the aptamer in the presence of the target also bring more polyanionic nucleic acids close to the surface, which contributes to greater resistance [21].

Over the last decades, nanotechnology has been introduced to analytical methods and sensing technologies to improve the sensitivities of platforms. In this direction, metallic nanomaterials such as iron [22], zinc [23,24], nickel [9,25], and tin [18,26] have been suggested as promising matrices for the sensing application. Tin nanoparticles (SnNPs) are especially promising materials that exhibit unique physicochemical properties to promote transduction processes in electrochemical sensors. The electrical and thermal properties of this group IV transition metal depend upon their size and morphology. In bulk Sn metal, electronic energy levels are distributed to form quasi-continuum bands, which are further replaced with the quantum confinement effect, and bands with discrete levels are generated when the Sn size is reduced to the nano range. Additionally, the cubic or tetragonal crystal phase of SnNPs provides a combination space for biological recognition molecules, such as aptamers [27,28]. Furthermore, the high electronic conductivity [29] and high specific capacity [30] of SnNPs will improve electronic machinery in electrochemical sensing technologies. To date, several studies have been reported on SnO_2_-based electrochemical sensors for small metal detection [31,32], but no studies have been published on SnNPs-based electrochemical aptasensors in biomedical diagnostics.

Electroanalytical techniques have become indispensable tools in modern analytical chemistry, and electrochemical methods have also been used for determining amphetamine-type substances (ATS). We recently designed an aptamer-modified gold nanoflower (AuNF)-based electrochemical aptasensor and demonstrated its sensitivity for amphetamine detection in spiked urine samples [33]. Following a similar concept, an aptamer-based electrochemical sensor for MDMA detection was designed in the present study, in which SnNPs-modified electrodes were used as an immobilization platform for a previously described aptamer with an affinity for ATS (referred to here as *Apt_MDMA_*). For successful fabrication of *Apt_MDMA_* on an SnNPs-based gold electrode (SnNPs@Au), the platform was functionalized with cysteamine (Cys) and glutaraldehyde (Glu) for covalent bonding between aptamers and the electrode surface. Subsequent to optimization and analytical validation, the aptamer-based sensor was applied in the analysis of biological specimens, such as spiked human urine, blood, and water. Limited studiesreporting on sensors for MDMA detection and none has utilized aptamers for MDMA identification. This is the first study to combine SnNPs and aptamers for the detection of illicit compounds.

## 2. Experimental Section

### 2.1. Chemicals and Reagents

The (±)-3,4-Methylenedioxymethamphetamine solution (MDMA; 1.0 mg/mL in methanol), Amphetamine (AMP; 1.0 mg/mL in methanol), 4-Hydroxybutyric acid sodium salt solution (GHB; 1.0 mg/mL in methanol), and Glu solution (50 wt% in water) were purchased from Sigma Aldrich, India. Additionally, N-Ethyl-N′-(3-dimethylaminopropyl)carbodiimide (EDC), N-hydroxysuccinimide (NHS), 2-(N-morpholino)ethanesulfonic acid (MES), 6-Mercapto-1-hexanol (6-MCH), potassium chloride (KCl), Tris, ethylenediaminetetraacetic acid (EDTA), anisole, benzaldehyde, potassium hexacyanoferrate (III) (K_3_Fe(CN)_6_), and potassium ferrocyanide (K_4_Fe(CN)_6_) were commercially obtained from SRL Pvt. Ltd., Mumbai, India. The EDC (2.0 mM)-NHS (5.0 mM) solution was freshly prepared in 100 mM MES solution (pH 5.0) at room temperature (24 ± 3 °C).

### 2.2. Apparatus and Procedures

Data acquisition for electrochemical measurements was performed with EC-Lab V11.10 software on a Biopotentiostate workstation (BioLogic science Instrument, Thane West, India, model no. SP 150). This three-electrode cell system was used to execute Cyclic Voltammetry (CV), Electrochemical Impedance Spectroscopy (EIS), and Differential Pulse Voltammetry (DPV). SnNPs were synthesized as per the literature. Morphological characterization was obtained by Scanning Electron Microscopy (SEM) with Energy-Dispersive X-ray (EDX) spectroscopy at Ozone Scientific, Bengaluru, India. Additionally, a High-Resolution Field-Emission Scanning Electron Microscope (FESEM; NOVA NANOSEM-450,FEI, GG Eindhoven, The Netherlands) was used at Jamia Millia Islamia University, New Delhi, to measure the stepwise fabrication of the electrode. X-ray diffraction (XRD; D2 Phaser, Brukers, Billerica, MA, USA) and Fourier transform infrared spectroscopy (FTIR; Nicolet iS5, Thermo Scientific, Waltham, MA, USA) were used to evaluate the structural properties of the nanoparticles at Amity University Uttar Pradesh (AUUP), Noida, India. X-ray photoelectron spectroscopy (XPS; PHI5000 Version Probe III, ULVAC-PHI, Inc., Osaka, Japan) of SnNPs was conducted at SRM University, Chennai, India.

### 2.3. Synthesis and Characterization of Tin Nanoparticles (SnNPs)

The SnNPs (~80 nm) used in this study were prepared using the electrical explosion method, as previously reported in our laboratory [34]. For morphological characterization, SEM was performed at 25 kV beam energy. The XRD-based characterization of SnNPs was performed at a 1.54 Å wavelength of Cu K-α at a scan rate of 1°/min. The crystal size (*D*) was calculated with Debye–Scherrer’s equation, which is:

(1) $$D=\frac{K\lambda }{\left(\beta cos\theta \right)},$$

where *K* is the dimensionless Scherrer shape constant (0.94), *λ* is the wavelength (1.54 Å), *β* is the full width at half maximum (FWHM), and *θ* is the Bragg angle. Further elemental analysis of the nanoparticles was determined by EDX using silicon drift detectors (SDD) at 25 kV beam energy. The functional group and coordination of the SnNPs were analyzed via FTIR in the frequency range of 500–4000 cm^−1^. Samples for XPS studies were prepared by slow evaporation of a nanomaterial suspension deposited on a 1 cm^2^ silicon support. The spectra were recorded with Al mono radiation of 55 eV as the binding energy.

### 2.4. Preparation of Apt_MDMA_Solutions

The amino-functionalized aptamer, which we refer to here as *Apt_MDMA_* [35], was synthesized and HPLC-purified by Integrated DNA Technologies (IDT), USA, with a sequence of 5′-(NH_2_)-(CH_2_)_6_-ACGGTTGCAAGTGGGACTCTGGTAGGCTGGGTTAATTTGG-3′. Although the secondary structure of *Apt_MDMA_* has not been reported, we note the existence of consecutive guanosine residues (underlined) that could potentially form a guanosine quadruplex. Solutions of 0.5, 1.0, 1.5, and 2.0 μM *Apt_MDMA_* were prepared in 1X TE buffer (10 mM Tris-HCl, pH 8.0, 1 mM EDTA) and stored at 4–8 °C until further use.

### 2.5. Electrochemical Characterization of Apt_MDMA_/SnNPs@Au

The gold electrode was cleaned with a solution of H_2_SO_4_ and H_2_O_2_ (*v*/*v* 3:1) for 30 min and washed with distilled water. Then, the electrode was immersed in water and ethanol solution (1:1 ratio) for at least 20 min. Before SnNPs deposition, the nanomaterial was oxidized to tin ions by mixing them with 1.0 M HCl solution, and the sample was vacuum oven-dried for 90 min at 70 °C. Later, the sample was allowed to come at the room temperature and was scraped to collect SnNPs in powder form. The dried SnNPs were mixed with 0.25 M NaCl and ultra-sonicated for 15–20 min to disperse the nanoparticles for use as electrolytes for electrode fabrication. The electrodeposition of oxidized SnNPs on a gold electrode was optimized via Chronocoulometry (CC), which was performed at −0.1 V for 15, 30, and 45 min. Finally, the redox potential was studied between −0.5 V and 0.5 V at a scan rate of 100 mV/s, and the EIS study was performed in a frequency range of 1.0 MHz–500 MHz in 5.0 mM [Fe(CN)_6_]^3−/4−^ electrolyte in the presence of 0.1 M KCl.

The *Apt_MDMA_*/SnNPs@Au-based sensor in our study was prepared through the following steps. First, the prepared SnNPs@Au electrode was submerged in 1.0 mM Cys at 4–8 °C overnight to place –NH_2_ groups on the surface. Later, the modified electrode was allowed to incubate in 2.5% Glu solution for 1 h, followed by the activation of carboxylic groups after treatment with freshly prepared EDC (2.0 mM)-NHS (5.0 mM) solution for another 1 h at room temperature. After this, the EDC-NHS/Cys/SnNPs@Au electrode was incubated in varying concentrations of *Apt_MDMA_* (0.5, 1.0, 1.5, and 2.0 μM) to optimize the aptamer concentration. *Apt_MDMA_* was allowed to deposit onto the modified electrode via the drop-cast method at 4.0 °C overnight. Later, *Apt_MDMA_*/SnNPs@Au was incubated in 10 μM 6-MCH for 1 h, which displaced nonspecifically adhered *Apt* to achieve a well-aligned oligonucleotide monolayer. Finally, electrochemical transduction of *Apt_MDMA_*/SnNPs@Au was studied by CV and EIS with a potential between −0.5 V and 0.5 V at a scan rate of 100 mV/s and a frequency range of 1.0 MHz–500 mHz, respectively, in 5.0 mM [Fe(CN)_6_]^3−/4−^ electrolyte in 0.1 M KCl.

To determine the effective surface area of the electrode, the electrochemical response of bare Au, SnNPs@Au, and *Apt_MDMA_*/SnNPs@Au electrodes were evaluated at scan rates of 20–100 mV/s. As per the Randles–Sevcik equation for electrochemical processes [36,37], the electrochemically accessible surface area of the electrode was calculated via the following equation:

(2) $$I\;\left(mA\right)=\left(2.69\times 10^{5}\right)A\cdot \sqrt{D}\cdot \sqrt{n^{3}}\cdot \sqrt{v}\cdot C,$$

where *I* is anodic peak current (*mA*), *A* is the electroactive surface area of the electrode (cm^2^), *D* is the diffusion coefficient (7.2 × 10^−6^ cm^2^/s for [Fe(CN)_6_]^3−/4−^ in 0.1 KCl solution [38]), *n* is the number of electrons transferred in the redox event (generally 1), *C* is the concentration of electrolyte (mol/cm^3^), and *ν* is the scan rate (mV/s), which was varied from 20–100 mV/s. Electrochemical measurements were obtained via CV from a potential range of −0.5 to +0.5 V in 5.0 mM [Fe(CN)_6_]^3−/4−^ electrolyte in the presence of 0.1 M KCl solution.

### 2.6. Determination of Aptasensor Response to MDMA Analyte

To measure the efficiency of the *Apt_MDMA_*/SnNPs@Au electrode, the fabricated aptasensor was incubated with samples that contained 0.001, 0.01, 0.1, 0.2, 0.4, 0.6, 0.8, and 1.0 nM MDMA, followed by immersion in 5.0 mM [Fe(CN)_6_]^3−/4−^ electrolyte in the presence of 0.1 M KCl solution for Potentiostatic Electrochemical Impedance Spectroscopy (PEIS) in a frequency range of 1.0 MHz- 500 mHz (*Note: PEIS is a useful technique to measure impedance at different voltages in a given frequency range*). The limit of detection (*LOD*) and sensitivity of the sensor was calculated from the calibration curve obtained at different impedances. The *LOD* calculation was based on the standard deviation of the response (*Sy*) of the curve and the slope of the calibration curve (*S*) at levels approximating the LOD according to the formula:

(3) $$LOD=3.3\left[\frac{S_{y}}{S}\right],$$

On the other hand, sensitivity refers to the ratio of the output change Δ*R_ct_* to the input change Δ*MDMA* under steady-state operation, which is the slope of the output-input characteristic calibration curve. For the aptasensor described in this study, sensitivity was calculated by the following formula:

(4) $$Sensitivity=\frac{\Delta R_{ct}}{\Delta MDMA\;conc.\;\;\left(nM\right)},$$

The incubation time of the MDMA sensor was also optimized by allowing the target MDMA to bind with the *Apt_MDMA_*/SnNPs@Au electrode for 5, 15, 30, 45, 60, 75, 90, 105, or 120 min. Then, PEIS was performed in the frequency range of 1.0 MHz–500 mHz in 5.0 mM [Fe(CN)_6_]^3−/4−^ electrolyte in a 0.1 M KCl solution. The aptasensor was regenerated after each step of target binding by rinsing with 6.0 M urea solution with continuous stirring for 10 min at 35 °C. 

### 2.7. Optimization of Analytical Parameters

The effect of pH on the analytical performance of the *Apt_MDMA_*/SnNPs@Au electrode was evaluated at pH values of 4.5, 5.0, 5.5, 6.0, 6.5, 7.0, 7.5, 8.0, 8.5, 9.0, and 9.5 in the presence of 0.1 PBS (137 mM NaCl, 2.7 mM, 10 mM Na_2_HPO_4_, and 1.8 mM KH_2_PO_4_). PEIS was recorded in the frequency range of 1.0 MHz–500 mHz in 5.0 mM [Fe(CN)_6_]^3−/4−^ and 0.1 M KCl electrolyte solution. Furthermore, the selectivity of the developed electrochemical aptasensor was assessed by evaluating the electrochemical response in the presence of interferents, including 0.33 nM AMP, GHB, aspirin, benzaldehyde, benzoic acid (BA), and aniline. Furthermore, the stability of the developed aptasensor was also measured over a month, during which DPV was performed under optimum conditions once per week. The precision and reproducibility of the developed aptasensor were evaluated via inter- and intra-batch studies, in which the electrochemical response of the *Apt_MDMA_*/SnNPs@Au electrode was recorded every 2 h for 10 h (a total of six times) or at fixed times on alternate days for six successive measurements.

### 2.8. MDMA Detection in Real Samples

The efficacy of the developed aptasensor was evaluated by measuring the electrochemical analytical performance of the *Apt_MDMA_*/SnNPs@Au electrode in the presence of spiked biological and water samples. Four human urine and blood samples were obtained from the Biodiagnostic Lab., East Rohini, New Delhi, India, and stored at −20 °C before use. Urine samples were used within one day of being received. Particulate matter was removed from urine samples via a 5.0 μm filter syringe, and the filtrate was further diluted 100 times with 0.1 M phosphate buffer (75.4 mM Na_2_HPO_4_ and 24.6 mM NaH_2_PO_4_; pH 7.4) and equilibrated for 30 min at room temperature. Then, 0.1 mL of each processed urine sample was spiked with MDMA to a final concentration of 0.1, 0.4, 0.7, or 1.0 nM, and electrochemical responses were recorded via PEIS in a frequency range of 1.0 MHz to 500 MHz in 5.0 mM [Fe(CN)_6_]^3−/4−^ and 0.1 M KCl solution. Signal responses were compared to those of control samples, which were urine samples that did not contain the MDMA analyte. Each sample was measured three times on *Apt_MDMA_*/SnNPs@Au electrodes, and the Relative Standard Deviation (RSD%) was calculated. Similar experiments were performed with spiked water samples to further evaluate the analytical performance of the developed aptasensor.

## 3. Results and Discussion

### 3.1. Strategy of Aptasensing for MDMA Detection

**Scheme 1:**
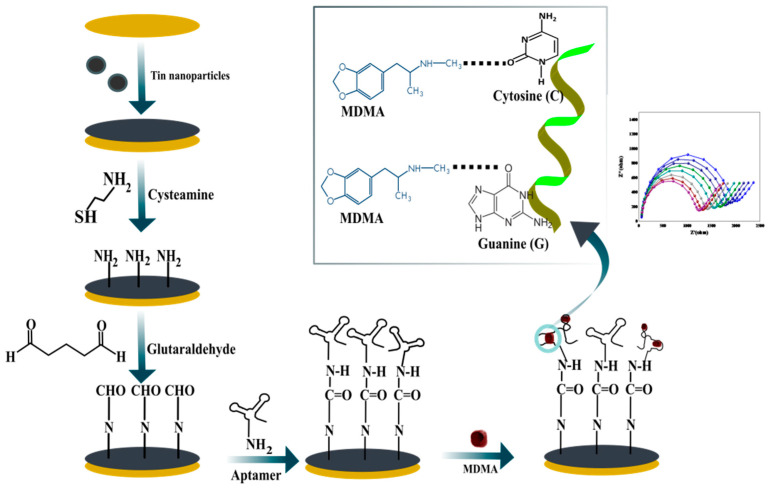
Schematic representation of electrode fabrication and electrochemical response for MDMA detection.

In this work, a novel electrochemical aptasensor was constructed for MDMA detection, as illustrated in the Scheme 1. Oxidized SnNPs were prepared and then electrochemically deposited onto the gold electrode. This approach combines the advantages of a high surface area for more aptamer deposition and an excellent electrochemical response to the target analyte [10,39,40]. Fabricated SnNPs were further functionalized with Cys to provide an NH_2_ group on the surface that reacts with the carboxylic group of Glu and ultimately provides a binding site for amino-functionalized *Apt_MDMA_*. *Apt_MDMA_* can anchor to the large surface area of the SnNPs-modified electrode through covalent attachment [21,41]. Based on extensive precedent from other aptamers, it is presumed that the *Apt_MDMA_* strands will change their conformation and flexibility upon noncovalently binding with MDMA. The conformational changes of aptamers are expected to alter the accessibility of the electrode surface, thereby affecting the current flow [42]. This ultimately results in reduced electron transfer between the modified electrode and electrolyte solution, which is the major determining factor in electrochemical sensing technologies. 

### 3.2. Characterization of SnNPs

**Figure 1:**
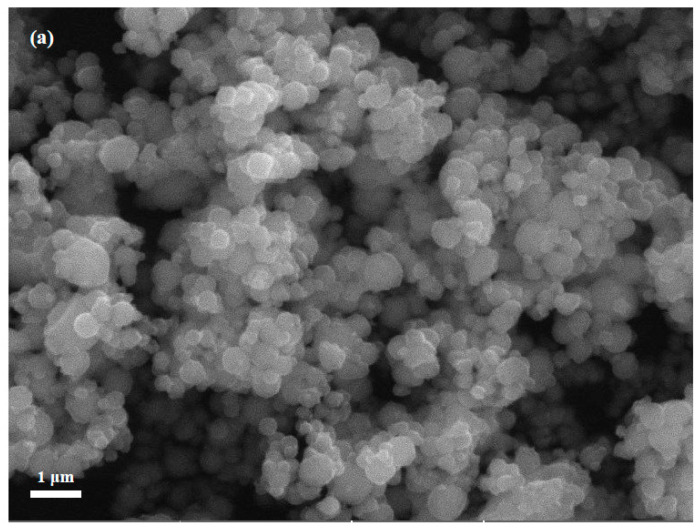

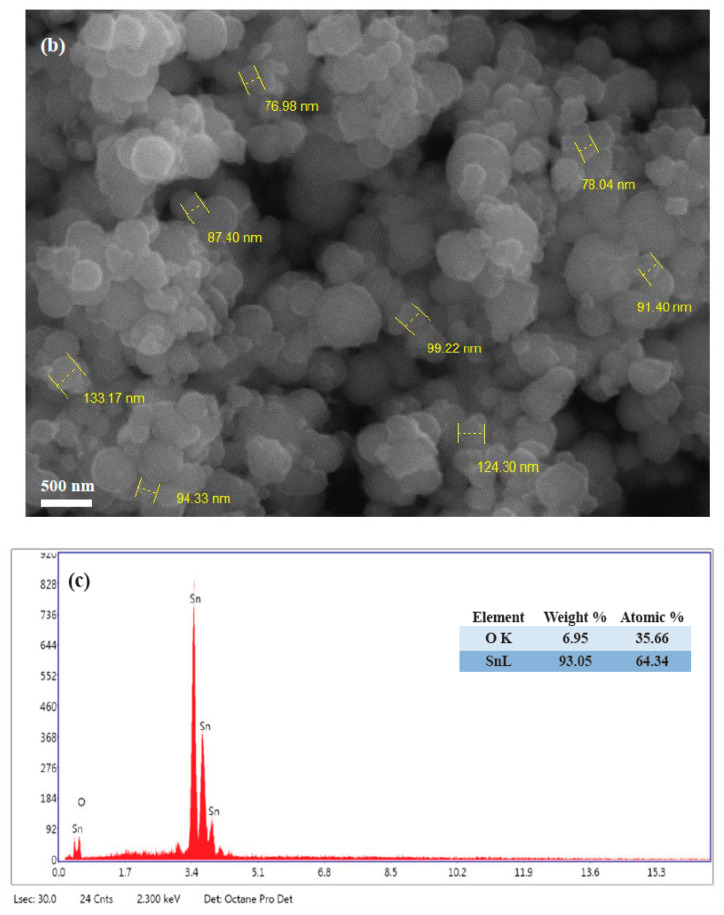
Scanning Electron Microscopy (SEM) imaging of prepared tin nanoparticles (SnNPs) at different resolutions: (**a**) 100 μm (**b**) 500 nm, and **(c)** Energy-Dispersive X-ray (EDX) spectra.

The morphology and surface structure of prepared SnNPs were analyzed by SEM, as shown in Figure 1a,b. The synthesized SnNPs were uniform in both size and shape with a mean diameter of ~60 nm, as determined from the crystal size analysis in XRD. The EDX spectra are also presented in the inset of Figure 1c, which confirm the presence of tin within the nanomaterial.

**Figure 2:**
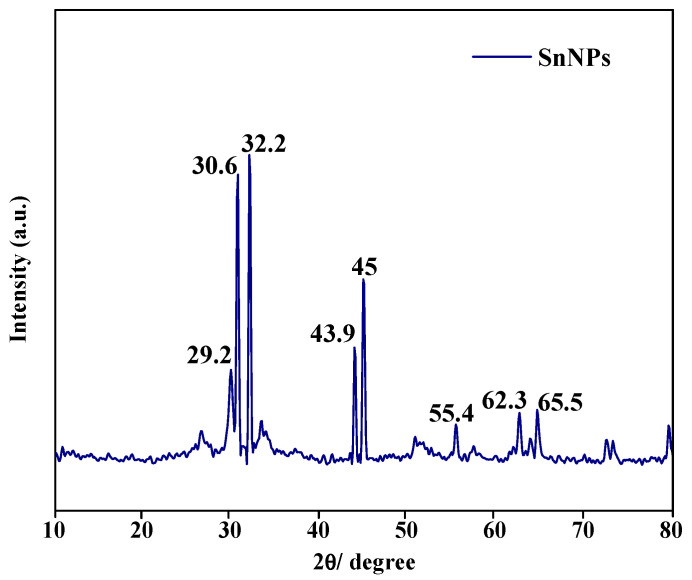
X-ray diffraction (XRD) image to evaluate physical and structural characteristics of synthesized tin nanoparticles (SnNPs).

An XRD analysis was performed to evaluate the crystalline properties and identify the prepared SnNPs. The XRD pattern in Figure 2 demonstrates diffraction peaks at 30.6, 32.2, 43.9, 55.4, 62.3, and 65.5°, corresponding to (101), (110), (200), (301), (103), and (321) planes, respectively, of tetragonal SnNPs [43,44,45] (Table 1). This result confirms that the samples prepared are indeed SnNPs with no crystal impurities. The average crystalline sizes were calculated via the Scherrer equation for the two most intense ((101) and (110)) planes and were found to be 53 nm and 37.5 nm, respectively, which supports the results obtained in SEM imaging.

**Figure 3:**
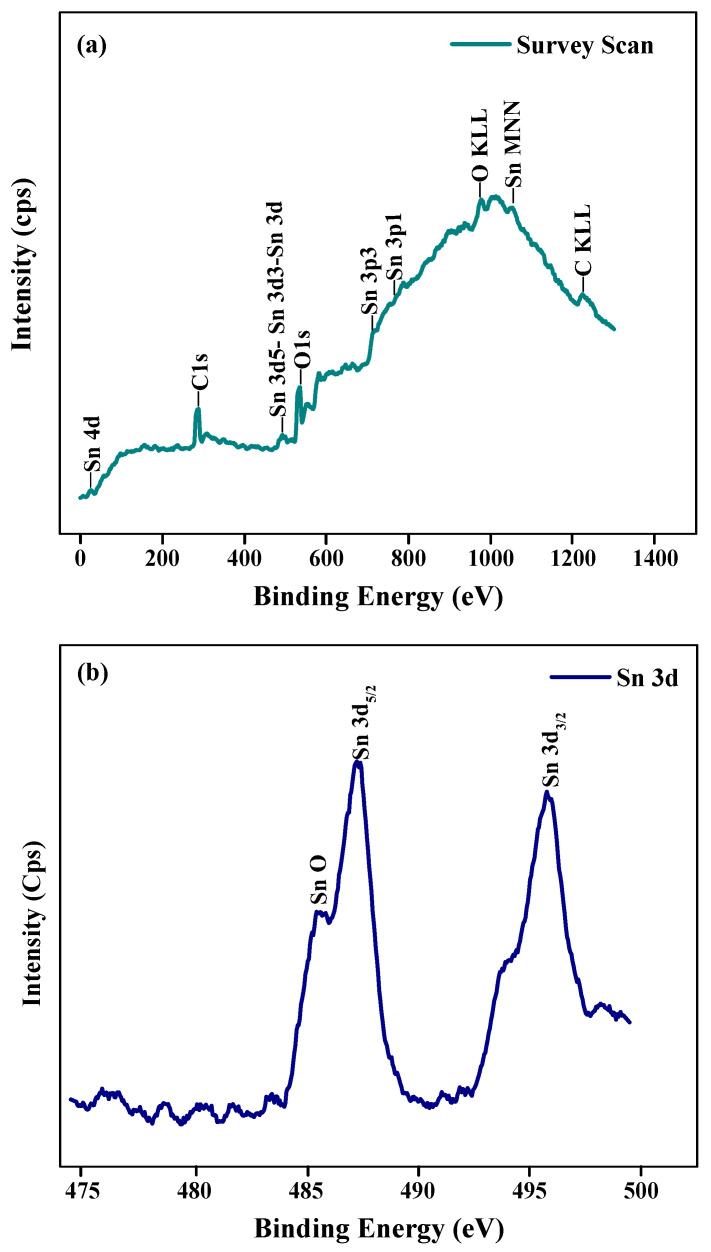
X-ray photoelectron spectroscopy (XPS) spectra of SnNPs surface: (**a**) survey scan and (**b**) high-resolution spectra of Sn 3d.

For further elucidation of the composition phase and chemical state of the prepared nanomaterial, X-ray photoelectron spectroscopy (XPS) was also performed to investigate its surface chemical state. The presence of Sn, O, and C elements was confirmed in the XPS data, as shown in Figure 3a, with high-resolution XPS spectra for Sn 3d_3/2_ and Sn 3d_5/2_ (Figure 3b). The Sn 3d_5/2_ and Sn 3d_3/2_ peaks were fitted by peaks at binding energies of 485.5, 487.2, and 494.3 eV, which correspond to Sn, Sn^+2^, and Sn^+4^ [46]. However, XPS data also exhibited two small peaks of O 1s at 533 and 973 eV, but FTIR data confirm a negligible amount of oxygen in the system. Thus, these peaks in XPS might be of oxygen present in the environment while performing XPS. In summary, our results appear to verify the purity of the prepared nanomaterial elements in the metallic state.

### 3.3. Electrochemical Characterization of Aptasensor

**Figure 4:**
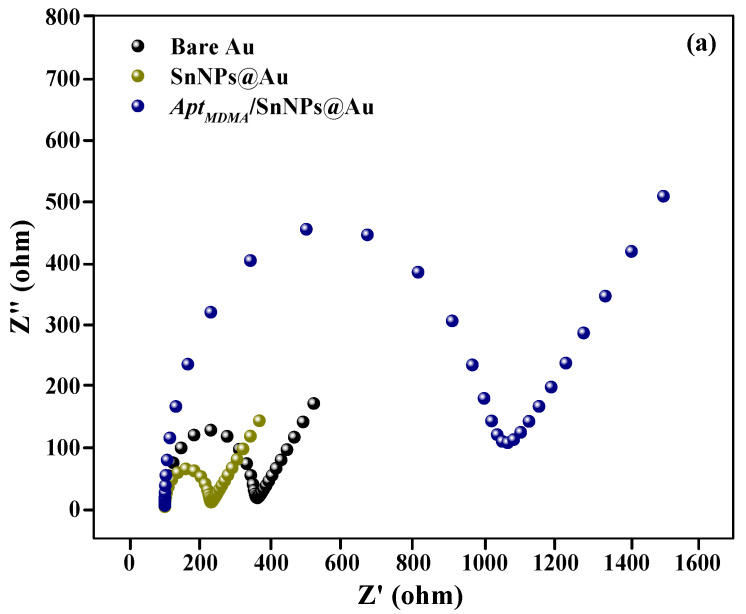

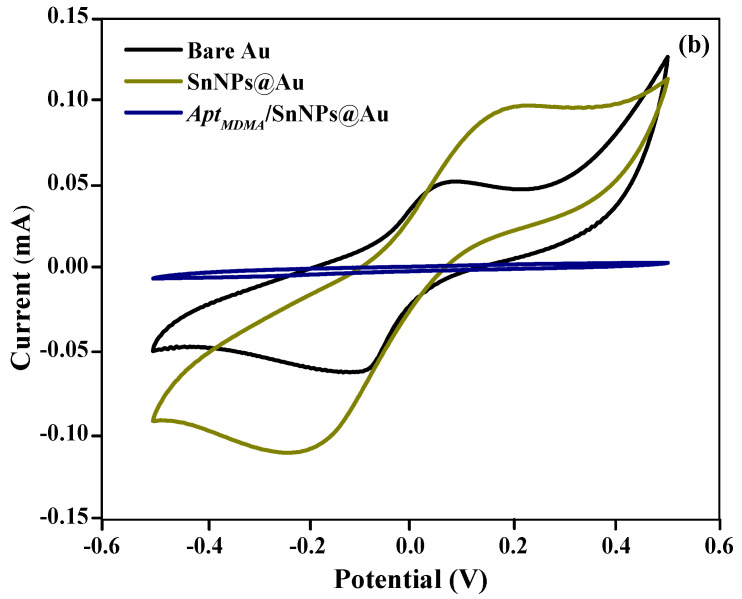
Electrochemical properties of tin nanoparticle (SnNPs)-based aptasensor monitored in 5.0 mM [Fe(CN)_6_]^3−/4−^and 0.1 M KCl electrolyte. (**a**) Electrochemical Impedance Spectroscopy (EIS) Nyquist plots of sequentially fabricated aptasensor for (±)-3,4-Methylenedioxymethamphetamine (MDMA) detection in the frequency range of 1.0 MHz–500 MHz, including bare Au electrode (black), SnNPs@Au (green), and *Apt_MDMA_*/SnNPs@Au (blue). (**b**) CV responses of the modified working electrode for target MDMA detection. Voltammograms are shown for bare Au electrode (black), SnNPs@Au (green), and *Apt_MDMA_*/SnNPs@Au (blue).

To prove the feasibility of biosensing with the aptasensor described above, PEIS and CV were used to study the interfacial properties of the electrode during the sequential stages of its fabrication. PEIS measures charge transfer resistance (*R_ct_*) values, which represent charge transfer kinetics in the absence of mass transfer limitation and are inversely proportional to the exchange current between the electrolyte and the sensing surface. Figure 4a illustrates the Nyquist graphs of the electrode during its stepwise modification. It shows that the impedimetric response decreased at SnNPs@Au (*R_ct_* = 226 Ω) compared to the bare electrode (*R_ct_* = 360.9 Ω), which further increased for the *Apt_MDMA_*/SnNPs@Au-modified electrode (*R_ct_* = 1.055 kΩ). This result indicates that SnNPs had the highest conductivity with a high surface area, which supports a dynamic balance of the Sn° = Sn^2+^ + 2e^−^ mechanism. The presence of Sn^2+^ and the induced electron contributing conductance in the system caused low *R_ct_* values [47,48]. On the other hand, the negative charges on the phosphodiester backbone of *Apt_MDMA_* caused the repulsion of redox species [49,50], thus reducing the redox reaction and enhancing the *R_ct_* value to 98.2% from the bare electrode. 

The variation in *R_ct_* values in Nyquist graphs is consistent with CV data in our study, as shown in Figure 4b. The bare electrode exhibited well-defined anodic and cathodic peak currents due to the reversible interconversion of the redox-active electrolyte [Fe(CN)_6_]^3−/4−^. The CV of an electrochemical system is characterized by the separation of forward and reverse peak potentials (Δ*E_p_*) to exhibit electron transfer kinetics between the electrode and the analyte, which determine the electrochemical reversibility of the system [51,52]. If the system is reversible, the analyte is stable upon reduction and can subsequently be reoxidized, a condition in which Δ*E_p_* should be more than 0.058 V. Similarly, the ratio of cathodic and anodic currents to achieve a stable redox system was also reported to be 1.0 [52]. Our study also exhibited a well-defined quasi-reversible redox voltammogram with a ratio of anodic and cathodic peak current (*I_a_/I_p_*) of ~1.0 and peak-to-peak separation ratio (Δ*E_p_*) of 0.439 V, hence confirming the development of a stable, reversible system. Following the electrodeposition of SnNPs onto the Au electrode, its reversibility was similar to that of the bare electrode, yet the peak current significantly exceeded that of the bare Au electrode. Furthermore, Δ*E_p_* between oxidation and reduction was also decreased to 0.177 V, indicating higher conductivity and improved electron transfer between the SnNPs@Au electrode and redox probe [53]. Once *Apt_MDMA_* was immobilized onto the SnNPs@Au surface, the electrode exhibited a significant decrease in peak current, consistent with the generation of a kinetic barrier between the negatively charged phosphate backbone of the aptamer and the [Fe(CN)_6_]^3−/4−^ electrolyte [54,55].

**Figure 5:**
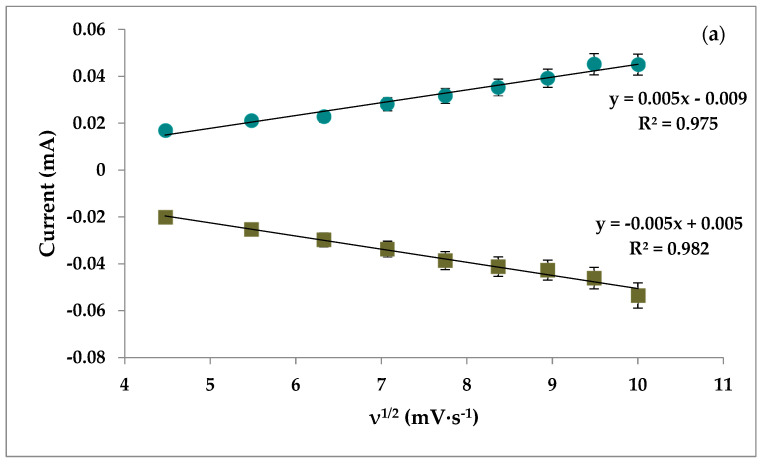

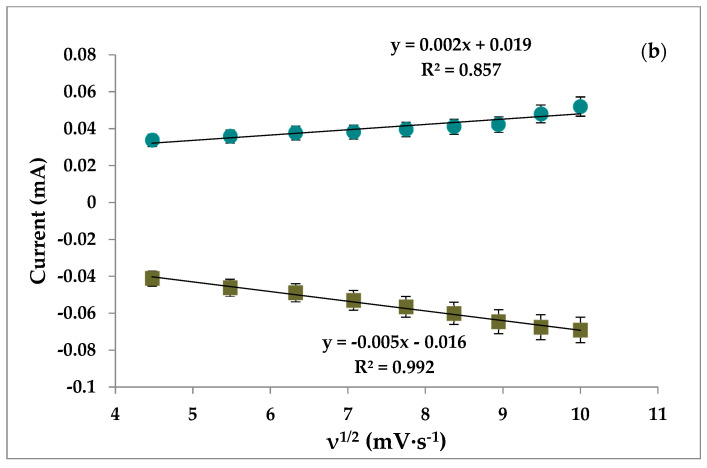
Calibration curve of redox peak and √scan rate for (**a**) bare Au electrode and (**b**) SnNPs@Au-modified electrode to determine electroactive surface areas.

The conducting properties of the SnNPs@Au-modified surface likely arise from its high surface area, which was calculated by the Randles–Sevcik equation. Figure 5 shows a linear relationship between redox current peaks and the square root of the scan rate of bare and SnNPs@Au-modified electrodes. As per Equation (2), the bare electrode has a surface area of 0.034 mm^2^, which increased ~40-fold after successful SnNPs electrodeposition. The large electroactive surface area of SnNPs@Au (1.42 mm^2^) not only improves the sensitivity of the sensor but also provides a large area for *Apt_MDMA_* immobilization [10,56].

**Figure 6:**
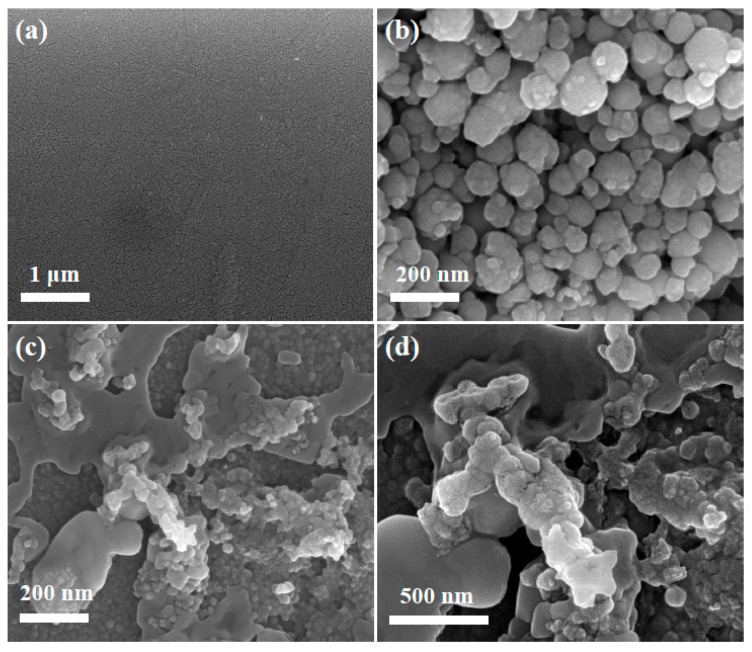
FESEM images of stepwise fabrication of electrode at different magnifications: (**a**) Bare (at 1 μm), (**b**) SnNPs (at 200 nm), (**c**) *Apt_MDMA_*@SnNPs (at 200 nm), and (**d**) *Apt_MDMA_*@SnNPs (at 500 nm).

The modification of the working electrode with SnNPs and the *Apt_MDMA_* probe was also confirmed by FESEM, as shown in Figure 6. The FESEM micrograph indicates that the morphology of SnNPs is spherical, yet the surface is rough (Figure 6b). This enhances the crystalline nature of the materials and thus provides a high electroactive surface area and large space for aptamer immobilization. Furthermore, Figure 6c,d indicates successful immobilization of *Apt_MDMA_* on the SnNPs surface.

**Figure 7:**
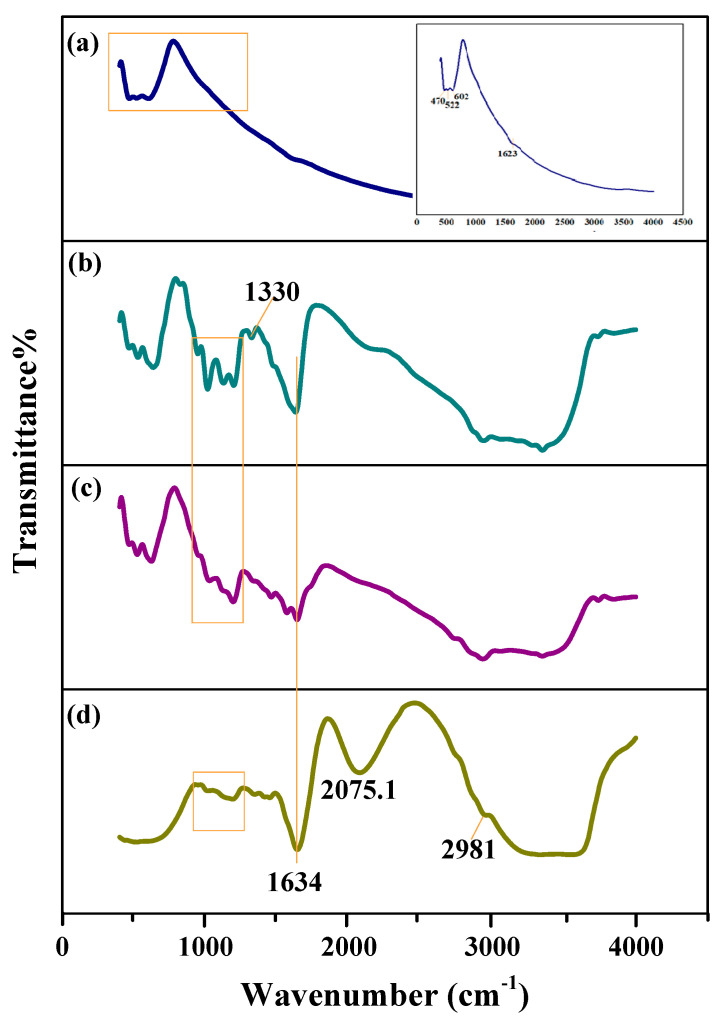
Fourier Transform Infrared Spectroscopy (FTIR) (500–4000 cm^−1^) spectra of electrode modification: (**a**) SnNPs. Inset shows FTIR spectra in the range of 400–1500 cm^−1^. (**b**) Cys@SnNPs, (**c**) Glu@Cys@SnNPs, and (**d**) *Apt_MDMA_*@Glu@Cys@SnNPs.

To demonstrate the process of electrode fabrication, we also performed FTIR at each step of electrode modification in the range of 500–4000 cm^−1^, as shown in Figure 7. The peaks at around 522 and 602 cm^−1^are assigned to the Sn-O stretching modes of Sn-OH and Sn-O-Sn, respectively [57], (Figure 7a). The small dip in the absorption peak at 1623 cm^−1^ is ascribed to hydroxyl group stretching, which indicates a negligible amount of surface-absorbed water [58,59]. Further fabrications with Cys@SnNPs and Glu@Cys@SnNPs are shown in Figure 7b,c. The peaks under the early fingerprint region, mainly 630 cm^−1^, are ascribed to the stretching vibration of C-S bonds on Cys@SnNPs, which confirms the first step of surface modification [60]. The peaks ranging between 1016 to 1199 cm^−1^ in Figure 7b show C = S stretching, corresponding to the thiocarbonyl group of Cys, while the other end containing the –NH_2_ group remains free for Glu attachment [61]. Furthermore, the peak at 1330 cm^−1^ corresponds to the C-H bending of Cys, which diminishes after Glu immobilization and confirms electrode modification. Another characteristic peak at 1717 cm^−1^ is also diminished in Figure 7c, which corresponds to the involvement of the –C = O bond of Glu with Cys. In addition, the peak at 1634 cm^−1^ corresponds to regeneration after –NH_2_-functionalized *Apt_MDMA_* immobilization on the Glu@Cys@SnNPs surface, which is explained by the overlapping of imino group absorption, formed by the reaction between the –COO^−^ group of Glu and the –NH_2_ group of *Apt_MDMA_* [62]. The characteristic peak in Figure 7d is at 2075.1 cm^−1^, which is the C≡C or C≡N stretching of nitrogenous bases of the aptamer, and thus confirms the *Apt_MDMA_* immobilization. Another peak at 2981 cm^−1^ represents the tetrahedral CH bond in the nucleotide, which is responsible for H-bonds among the *Apt* to maintain the structural integrity. Additionally, peaks in the range of 990–1011 cm^−1^ in Figure 7d correspond to the P-O-C stretching of aliphatic phosphates present in the phosphate group of *Apt_MDMA_*. Thus, these peaks confirm that SnNPs are linked to Cys with a thiol group, which is further modified with Glu via amide II bonds. This prepared Glu@Cys@SnNPs surface is functionalized with *Apt_MDMA_* via string covalent bonds between the crosslinker Glu and the –NH_2_ group of the *Apt*.

### 3.4. Optimization of Experimental Conditions

**Figure 8:**
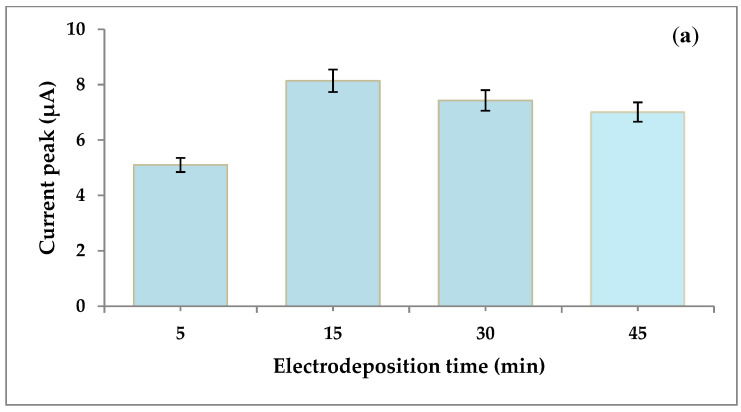

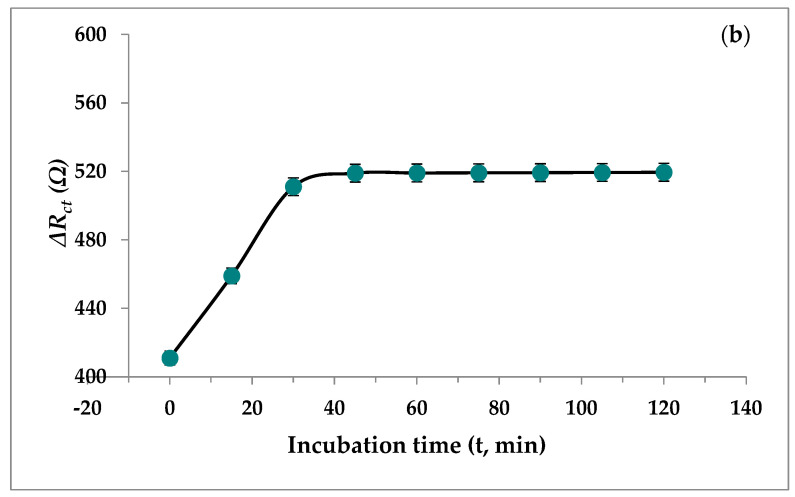

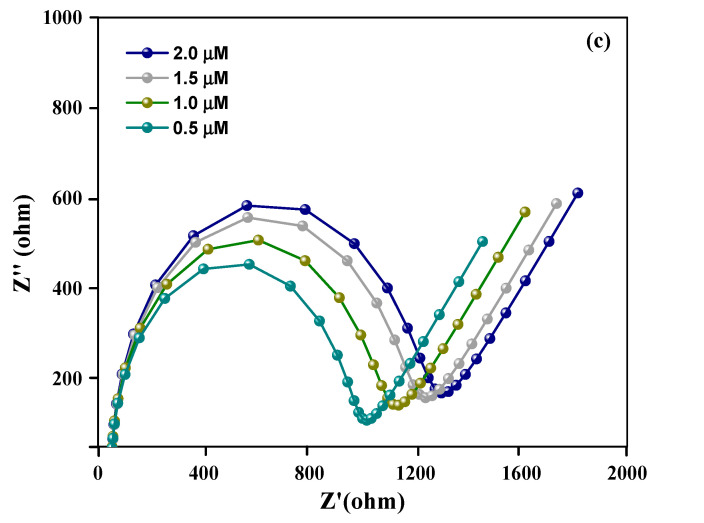

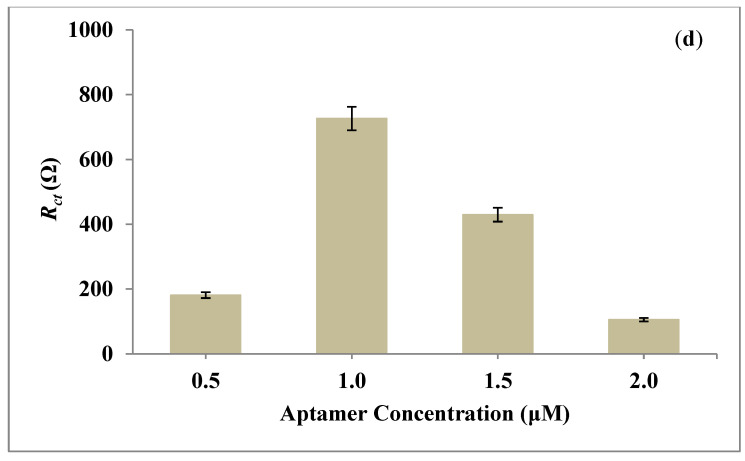
Optimization conditions to improve sensitivity of *Apt_MDMA_*/SnNPs@Au-modified electrode. (**a**) Bar graphs of Differential Pulse Voltammetry (DPV)-based peak currents at 5.0, 15, 30, and 45 min of SnNP electrodeposition. (**b**) Change in charge transfer resistance (Δ*R_ct_*) after incubation of MDMA on *Apt_MDMA_*/SnNPs@Au electrode for 0, 15, 30, 45, 60, 75, 90, 105, and 120 min. (**c**) Impedance Spectroscopy (EIS) Nyquist plots at different *Apt_MDMA_* concentrations (0.5, 1.0, 1.5, and 2.0 μM) in 5.0 mM [Fe(CN)_6_]^3−/4−^and 0.1 M KCl electrolyte. (**d**) Change in charge transfer resistance (Δ*R_ct_*) of 0.5, 1.0, 1.5, and 2.0 μM *Apt_MDMA_*-modified electrode in presence of (±)-3,4-Methylenedioxymethamphetamine (MDMA) analyte.

To improve the sensitivity and performance of the aptasensor, the electrochemical responses of SnNPs@Au were evaluated as a function of various parameters that are expected to affect its performance. First, the deposition time of SnNPs could significantly influence the conductivity of the sensor, as a prolonged time of SnNPs electrodeposition can potentially deform the fabricated layers due to clumping [63]. Additionally, the multi-layer formation of nanomaterials on the electrode surface via electrochemical nucleation and growth decreases the electrochemical reactivity of the sensor [64]. Therefore, the electrochemical responses of SnNPs@Au were measured after the electrodeposition of SnNPs for 5, 15, 30, and 45 min. As shown in Figure 8a, the peak current in DPV was highest for electrodes fabricated with 15 min of electrodeposition in comparison to 30 and 45 min electrodeposition. We suspect that the longer times of SnNPs deposition may promote dense surface clustering that modestly inhibits electron exchange between the electrode and the solution. 

The incubation time of the target with the *Apt_MDMA_*/SnNPs@Au electrode was also evaluated by incubating MDMA with the electrode for 5–120 min. As illustrated in Figure 8b, the Δ*R_ct_* values increased with time from 0 to 30 min and then held steady at longer times. Therefore, 30 min was considered the optimum incubation time in the current study. To evaluate the effect of *Apt_MDMA_* concentration on the sensitivity of the modified electrode, aptamer densities on the surface were calculated following the method reported by Liu et al. [65]. In the presence of 0.5, 1.0, 1.5, and 2.0 μM *Apt_MDMA_,* the surface densities of the aptamer were estimated to be (3.16 ± 0.17), (680 ± 360), (840 ± 110), and (900 ± 130) × 10^9^ molecules/cm^2^, respectively. As illustrated in Figure 8c, the *R_ct_* values increased with increasing *Apt_MDMA_* concentration (*Apt_MDMA_* probe density), which indicates a large quantity of negatively charged *Apt_MDMA_* on the surface. Δ*R_ct_* value was the maximum for the 1.0 μM *Apt_MDMA_*/SnNPs-modified electrode, with a lower signal for 1.5 μM *Apt_MDMA_* and little or no electrochemical response for 0.5 and 2.0 μM *Apt_MDMA_* (Figure 8d). Therefore, we selected 1.0 μM *Apt_MDMA_* in our study for further aptasensor development.

### 3.5. Analytical Performance of Aptasensor

**Figure 9:**
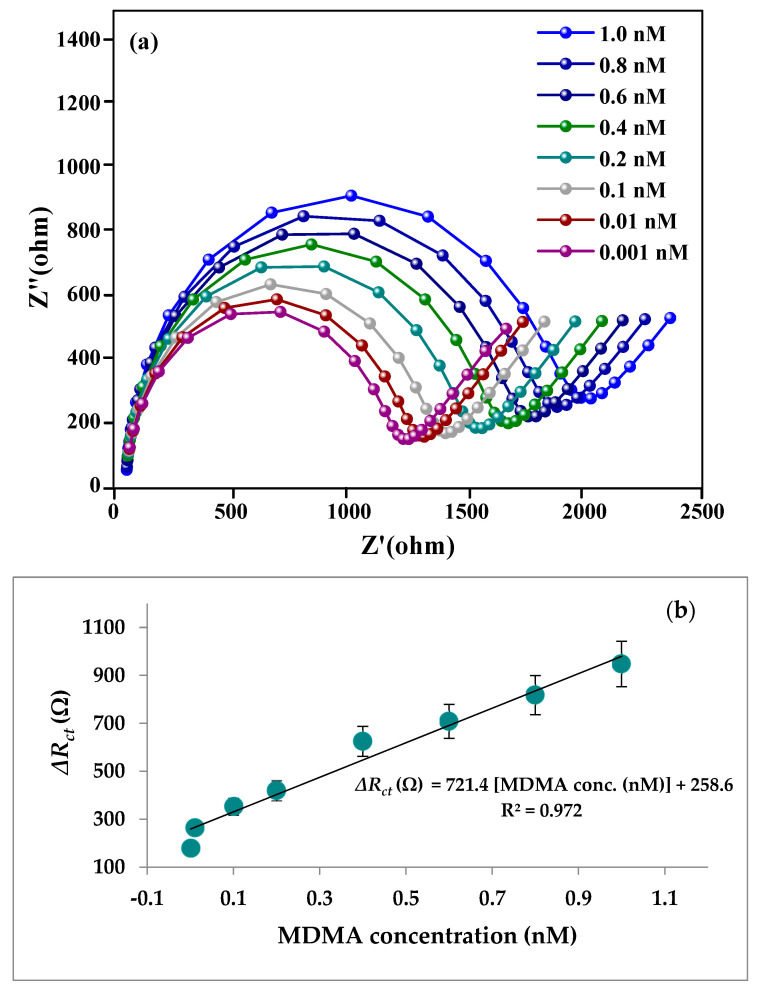
Performance analysis of *Apt_MDMA_*/SnNPs@Au electrode at different (±)-3,4-Methylenedioxymethamphetamine (MDMA) concentrations. (**a**) Change in charge transfer resistance (Δ*R_ct_*) at MDMA concentrations of 0.001, 0.01, 0.1, 0.2, 0.4, 0.6, 0.8, and 1.0 nM. Analyses were carried out in 5.0 mM [Fe(CN)_6_]^3−/4−^ and 0.1 M KCl electrolyte. (**b**) Calibration curves between Δ*R_ct_* and different MDMA concentrations.

The electrochemical sensing of the *Apt_MDMA_*/SnNPs@Au platform was tested using impedimetric measurements to investigate its ability and analytical performance in MDMA detection. As illustrated in Figure 9a, the impedance intensity of the aptasensor increases with increasing MDMA concentration from 0.001 to 1.0 nM as a result of efficient analyte capture by *Apt_MDMA_,* resulting in mass and electron transfer hindrance on the surface [66,67]. A linear relationship between Δ*R_ct_* (defined as Δ*R_ct_*= *R_ct, MDMA_ − R_ct, aptamer_*) and the MDMA concentration was observed in the range of 0.1–1.0 nM MDMA, as shown in Figure 9b, and can be expressed as a linear regression equation (Equation (5)) with a slope of 721 Ω/nM, a y-intercept of 259 Ω, and a correlation coefficient of 0.972. The limit of detection (LOD) of the sensor was calculated as 0.33 nM (defined as the analyte concentration at which signal/noise = 3) with a sensitivity of 0.54 Ω/nM.

(5) $$\Delta R_{ct}\left(\Omega \right)=721\left[\mathrm{MDMA}\;\mathrm{conc}.\;\left(\mathrm{nM}\right)\right]+258.6\;\left(R^{2}=0.97\right),$$

The above results establish that electrochemical amplification of MDMA detection was successfully achieved and that the SnNPs-based electroconductivity led to the effective sensitivity of the system, as expected. Our method compares favorably to other MDMA sensing strategies, as shown in Table 2.

### 3.6. pH and Scan Rate of Aptasensor

**Figure 10:**
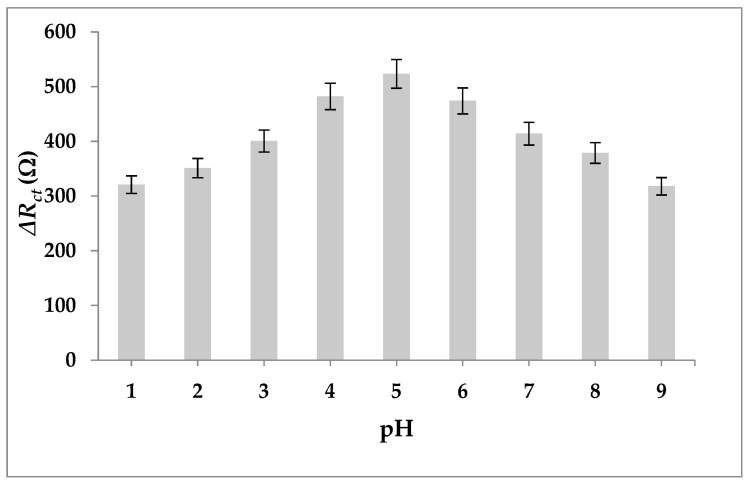
Effect of pH on analytical performance of the aptasensor in the presence of 0.33 nM MDMA.

To evaluate the effective pH for the efficient function of the aptasensor, PEIS data were recorded for the *Apt_MDMA_*/SnNPs@Au electrode in the presence of 0.33 nM MDMA in phosphate buffer at pH values ranging from 5.5–9.5. As shown in Figure 10, the aptasensor exhibited the highest impedance at pH 7.5, which indicates efficient functioning of the *Apt_MDMA_*/SnNPs@Au electrode near physiological pH; real-world measurements should include the pH adjustment of biological samples such as urine to approximately pH 7.5 to maximize sensitivity.

**Figure 11:**
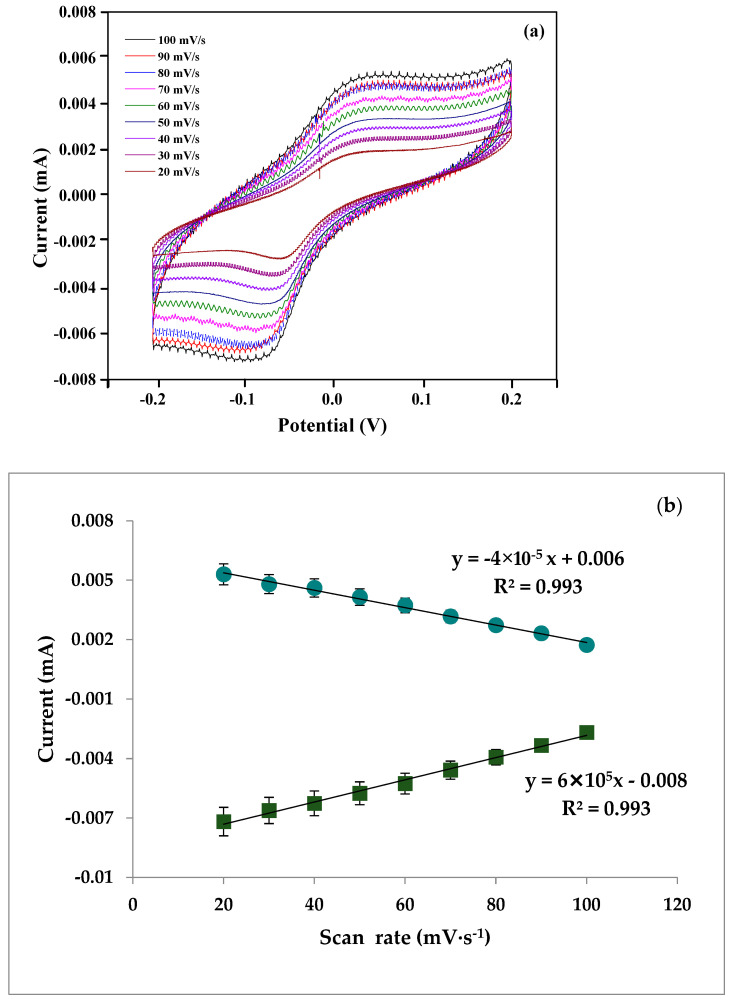
Determination of redox mechanism of aptasensor in 5.0 mM [Fe(CN)_6_]^3−/4−^ and 0.1 M KCl electrolyte in the presence of 0.33 nM. (**a**) Change in CV curves at scan rates of 20, 30, 40, 50, 60, 70, 80, 90, and 100 mV/s. (**b**) Calibration curves of redox peak current vs. scan rate.

The relationship between the scan rate and redox current peak was determined to shed light on the electrochemical mechanism of the *Apt_MDMA_*/SnNPs@Au electrode. When the peak current is proportional to the square root of the scan rate, then the process can be considered to be diffusion-controlled, while if it is linearly proportional to the scan rate, then it can be considered to be adsorption-controlled. Figure 11a shows the recorded voltammogram at scan rates of 20 to 100 mV/s in the presence of 0.33 nM MDMA. The CV graph shows that cathodic and anodic peak currents increased linearly with increasing scan rates. A scan rate of 50 mV/s was selected for subsequent experiments to obtain high sensitivity while minimizing the background noise of the current. The linear calibration curves between the redox peaks current (I) and scan rate, illustrated in Figure 11b, confirm an adsorption-controlled redox process in the sensor. The relation between the current and scan rate can be expressed as:

(6) $$I_{\mathrm{an}}\left(\mathrm{mA}\right)=6\times 10^{5}\left[\nu \;\left(\mathrm{mV}\cdot {\;s}^{-1}\right)\right]-0.008\;\left(R^{2}=0.993\right),$$

(7) $$I_{\mathrm{ca}}\left(\mathrm{mA}\right)=-4\times 10^{-5}\;\left[\nu \;\left(\mathrm{mV}\cdot {\;s}^{-1}\right)\right]-0.006\;\left(R^{2}=0.993\right),$$

### 3.7. Selectivity and Stability of the Aptasensor

**Figure 12:**
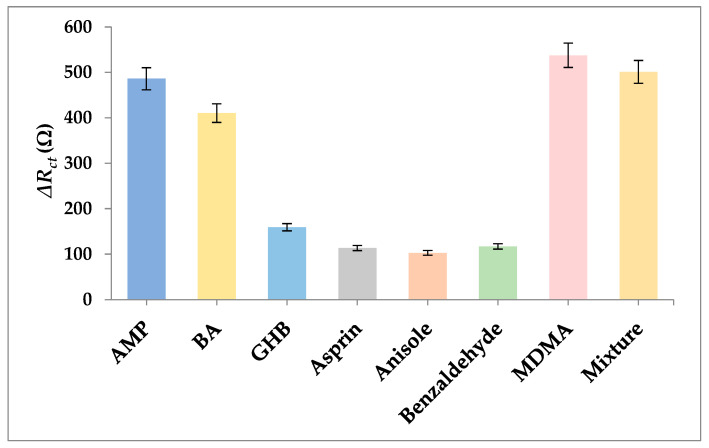
Selectivity analysis of aptasensor in the presence of 0.33 nM (±)-3,4-Methylenedioxymethamphetamine (MDMA), amphetamine (AMP), benzoic acid (BA), 4-Hydroxybutyric acid sodium (GHB), aspirin, anisole, benzaldehyde, and a mixture of the interferents.

To assess the specificity of the aptasensor for MDMA detection, Δ*R_ct_* was measured for potential interferents: AMP, GHB, aspirin, anisole, BA, and benzaldehyde, each at 0.33 nM. As illustrated in Figure 12, most of these potential interferents did not significantly perturb responses in comparison to the result obtained from MDMA. The single exception was AMP, whose chemical similarity to MDMA limits the selective detection of one compound to the exclusion of the other. The unavailability of potential major interfering recreational drugs, such as cocaine and ATS compounds such as heroin, preclude the evaluation of the selectivity of the aptasensor developed in our study with respect to these compounds. Moreover, the PEIS signal of the interferent mixture with MDMA was also similar to the current obtained in the presence of only MDMA, which indicates the potential selectivity of MDMA detection.

**Figure 13:**
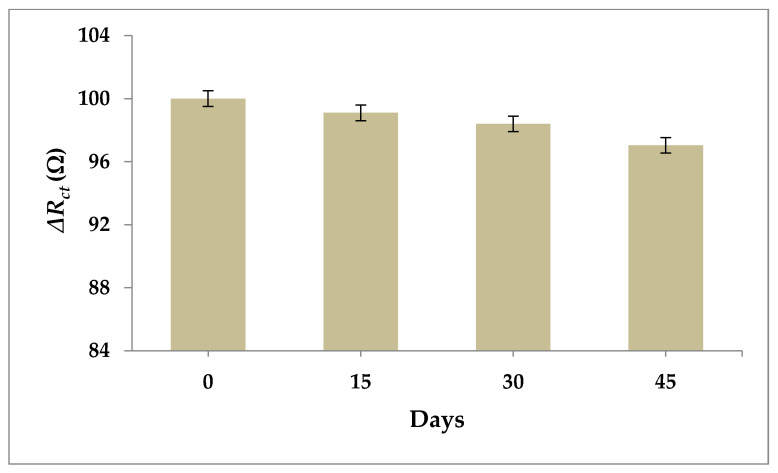
Stability analysis of aptasensor for (±)-3,4 Methylenedioxymethamphetamine (MDMA) detection.

The long-term stability of the aptasensor was evaluated by a storage assay, in which our sensor was stored at (4 ± 0.1) °C for 15 days, after which it retained 97% of its initial response (Figure 13). Furthermore, the impedimetric responses of *Apt_MDMA_*/SnNPs@Au in the presence of 0.33 nM MDMA were recorded for intra- and inter-batch studies, which confirmed the high reproducibility of the sensor with RSD% of 1.7% and <2.4%, respectively (Table 3). Thus, the experimental results suggest the acceptable selectivity, stability, and reproducibility of the aptasensor.

### 3.8. Performance of Aptasensor in Stimulated Real Samples

**Figure 14:**
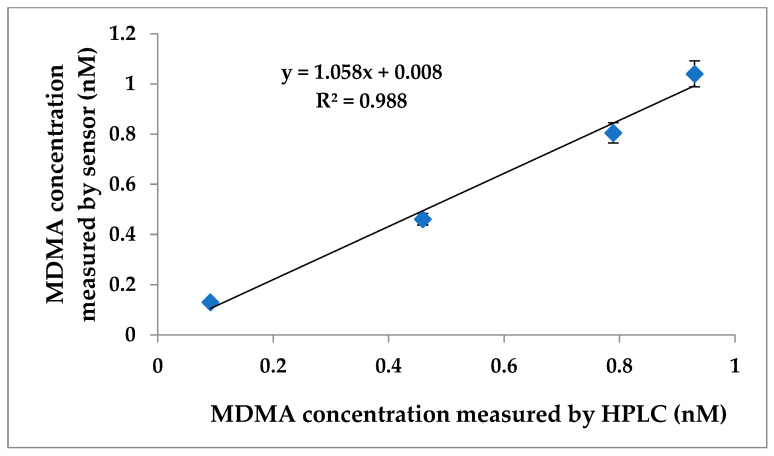
Correlation study of developed aptasensor compared to HPLC.

Finally, to demonstrate the utility of the aptasensor on complex samples, the aptasensor performance was evaluated on water and urine/ blood samples spiked with 0.1, 0.4, 0.7, or 1.0 nM of the MDMA analyte. PEIS measurements were carried out to evaluate signal recoveries and RSD% from the spiked samples, and the results are summarized in Table 4. The Δ*R_ct_* values before and after the addition of different concentrations of MDMA in real samples are close to the corresponding Δ*R_ct_* values in pure MDMA solution with the same concentrations. As exhibited in Table 4, the MDMA concentration recoveries ranged from 92–96.7%, 91–103%, and 87–90% for spiked urine, blood, and water samples, respectively with RSD values of 1.1–2.2% and 1.37–2.12% for urine and blood samples respectively. Additionally, a correlation study on the developed aptasensor also exhibited a high correlation (R^2^ = 0.98) with conventional HPLC (Figure 14). The excellent recovery percentages with low RSD values of the sensor suggest that the aptasensor developed here has good repeatability in real samples with potential application in forensic science.

## 4. Conclusions

In summary, we designed and implemented a simple and rapid electrochemical sensing strategy to detect MDMA in biological samples. The rapidly fabricated SnNPs play an important role in the sensitivity enhancement of the aptasensor in comparison to previously reported sensors. The high surface area of the SnNPs@Au-based electrochemical aptasensor provides ample space for the recognition element to bind, with optimal aptamer immobilization at 1.0 μM *Apt_MDMA_*. In addition, the aptasensor exhibited outstanding sensitivity, with a LOD of 0.33 nM and a sensitivity of 0.54 ohm/nM in a wide concentration range, with a linear response from 0.01 to 1.0 nM MDMA. Furthermore, it also exhibited its applicability to detect MDMA in spiked real samples for practical application. Moreover, the proposed sensor can access MDMA in urine samples for up to 4 h after ingestion of the drug; after that, MDMA metabolites, namely, 3,4-methylenedioxyamphetamine (MDA), 4-hydroxy- 3-methoxymethamphetamine (HMMA), and 4-hydroxy-3- methoxyamphetamine (HMA), are present in urine [72,73]. As a result, this useful sensing strategy enables the *Apt_MDMA_*/SnNPs-based sensor to be potentially applied in the areas of forensic science and analytical chemistry.

## Figures and Tables

**Table 1:** Crystalline size estimation of elemental SnNPs calculated using Scherrer equation.

SN	2θ (Degree)	d (A°)	FWHM (Degree)	Crystalline Size (nm)	Dislocation Density (δ)	Microstrain (ε)
1.	29.9	1.98	0.5538	25.2	0.157	0.037
2.	30.6	2.03	0.263	53.0	0.035	0.179
3.	32.2	2.12	0.371	37.5	0.071	0.026
4.	43.9	2.87	0.403	33.3	0.090	0.040
5.	45.0	2.95	0.307	43.2	0.053	0.032
6.	55.4	3.57	0.407	31.4	0.101	0.534
7.	62.3	3.98	0.369	33.5	0.089	0.055
8.	65.5	4.16	0.465	26.1	0.146	0.074

**Table 2:** Comparison of reported electrochemical sensors for MDMA detection.

SN	Electrochemical Sensor Type	Sensing Mechanism	Detection Limit	Linear Range	Incubation Time	Samples	Reference
1.	Cucurbit[6]uril-based sensor	CV	3.5 and 2.7 μM	4.2 × 10^−3^–4.8 × 10^−2^ μM	NR	NR	[5]
2.	Graphite-based sensor	DPV	40 μM	500–4980 μM	NR	PBS buffer	[68]
3.	Microcantilever-based immunosensor	Frequency shift	5.0 × 10^3^μM	5.0 × 10^3^–50 × 10^3^μM	NR	NR	[69]
4.	Gold electrode-based sensor	SWV	NR	110.9–258.9 μM	NR	Urine	[70]
5.	MIP-based sensor	SWV	0.7 μM	2.5–200 μM	10 min	Serum and urine	[71]
6.	Tin nanoparticle-based aptasensor	PEIS	0.33 nM	0.01–1.0 nM	30 min	Diluted blood, urine, and water	Present study

NR: Not reported.

**Table 3:** Precision of repeatability determination via intra- and inter-batch analysis of developed aptasensor.

	Mean Δ*R_ct_*	Standard Deviation	Coefficient of Variance (CV)
Intra-batch	541.97	9.28	1.71%
Inter-batch	546.98	13.2	2.42%

**Table 4:** Evaluation of *Apt_MDMA_*/SnNPs@Au-based sensing system for MDMA detection in spiked urine, blood, and water samples.

Added MDMA Conc. (nM)	Observed MDMA Conc. (nM)			Recovery %			RSD % (n = 3)		
	Urine	Blood	Water	Urine	Blood	Water	Urine	Blood	Water
0.0	ND	ND	ND	0.0	0.1	0.0	0.0		-
0.1	0.094	0.091	0.087	94%	91%	87%	1.79%	1.71%	-
0.4	0.384	0.380	0.354	96.7%	95%	88.5%	2.04%	1.99%	-
0.7	0.67	0.651	0.612	95.7%	93%	87.4%	2.18%	2.12%	-
1.0	0.92	0.923	0.901	92%	100.3	90.1%	1.14%	1.37%	-

## Data Availability

The data presented in this study are available within the article.
